# MicroRNA-582-5p suppresses non-small cell lung cancer cells growth and invasion via downregulating NOTCH1

**DOI:** 10.1371/journal.pone.0217652

**Published:** 2019-06-06

**Authors:** Jianghong Liu, Shengshuo Liu, Xiaoyan Deng, Jiaoyu Rao, Kaiyuan Huang, Gengrui Xu, Xiaokang Wang

**Affiliations:** 1 Department of Pharmacy, Shenzhen Longhua District Central Hospital, Shenzhen, China; 2 School of Pharmacy, Henan University, Kaifeng, Henan, China; 3 Department of Endocrinology, Shenzhen Longhua District Central Hospital, Shenzhen, China; 4 Department of Pharmacology, College of Pharmacy, Jinan University, Guangzhou, China; Universitat des Saarlandes, GERMANY

## Abstract

Non-small cell lung cancer (NSCLC) is the most common cancer worldwide. MicroRNAs have been shown to be correlated with biological processes of various tumors. In this study, we observed that the expression of miR-582-5p was lower in NSCLC tissues than that in para-carcinoma tissues. Ectopic expression of miR-582-5p significantly inhibited NCI-H358 cell proliferation and invasion. Knockdown of miR-582-5p showed the opposite results, with cell growth rate and the invasive capacity of PC-9 cells enhanced. Furthermore, we elucidated that NOTCH1 is a target of miR-582-5p and there is an inverse correlation between miR-582-5p and NOTCH1 expression in NSCLC tissues. Overexpression of NOTCH1 in miR-582-5p-overexpressing NCI-H358 cells could partially reverse the inhibition of cell proliferation and invasion by miR-582-5p. Thus, our research demonstrated that miR-582-5p suppresses NSCLC cell lines’ growth and invasion via targeting oncoprotein NOTCH1 and restoration of miR-582-5p might be feasible therapeutic strategy for NSCLC.

## Introduction

Lung cancer is the main cause of cancer-associated mortality all around the world and every year, about 1.6 million people die of lung cancer[1]. Non-small cell lung cancer (NSCLC) and small cell lung cancer (SCLC) are two subtypes of lung cancer. NSCLC, including adenocarcinoma, squamous cell carcinoma and large cell carcinoma, makes up approximately 85% of all lung cancer cases. Although technologies and strategies for treating NSCLC have improved in recent years, the prognosis of this disease is still poor. The 5-year survival rate of NSCLC is just around 15%[2]. It has been reported that tissue levels of specific microRNAs is associated with the pathological development of different cancers and overwhelming evidences have indicated that microRNAs can serve as potential diagnostic and prognostic biomarkers for various types of cancer[3, 4]. Therefore, searching new biomarkers and elucidating the underlying mechanism are fundamental for the development of new therapeutic treatments in NSCLC.

MicroRNAs (miRNAs) are a class of small noncoding RNAs, consisting of 19–24 nucleotides. They induce mRNAs degradation or translational repression through complementary base pairing with 3’-untranslated regions (3’UTR) of their target mRNAs[5, 6]. Based on previous studies, miRNAs have been widely proposed as potential targets for treating various cancers. For instance, miR-145 plays the proapoptotic and antiproliferative roles in colon carcinoma and miR-33a has capacity to suppress oncogenic kinase Pim-1. A.F et al showed that polyethylenimine (PEI)-mediated delivery of unmodified miR-145 and miR-33a are efficacious in a model of colon cancer[7]. J.K et al offered a preclinical proof that knockdown of an oncogenic microRNA miR-221 can block hepatocellular carcinoma growth and increase mouse survival[8]. Previous studies demonstrated that miR-582-5p exerts dual function against different kinds of tumors. For example, Wang et al[9] proved that miR-582-5p inhibits invasion and migration of salivary adenoid cystic carcinoma cells. Study by Zhang et al[10] showed that upregulation of miR-582-5p inhibits cell proliferation, cell cycle progression and invasion in human colorectal carcinoma. But in prostate cancer, Maeno et al verified that up-regulation of miR-582-5p contributes to an increase in cell proliferation under androgen deprived conditions[11]. However, it is unclear whether miR-582-5p plays a role as a tumor suppressor gene or an oncogene in NSCLC.

The Notch signaling pathway, a highly evolutionally conserved signal transduction network, is important for cell-fate determination and differentiation[12, 13]. Mammalian genome includes 4 NOTCH genes, of which NOTCH1 has been found to be aberrant activation in about 10% of NSCLCs. Due to lack of the expression of Numb gene, a negative regulator of Notch, the activity of NOTCH1 is increasing in another 30% of NSCLCs[14]. Preclinical studies described that activated NOTCH1 gets involved in tumorigenesis, proliferation and survival of NSCLC models through collaborating with Myc or modulating the expression of epidermal growth factor receptor (EGFR)[15, 16]. In addition, NOTCH1 has been reported to serve as a target of various miRNAs[17–20]. Given NOTCH1 is crucial for the development and progression of NSCLC and closely implicated with miRNAs, it is necessary to explore which miRNAs regulates NOTCH1 in NSCLC. Here, we clarified that miR-582-5p inhibits cell proliferation and invasion via decreasing the expression of NOTCH1 in NSCLC.

## Materials and method

### Clinical specimens

A total of 30 matched NSCLC tissues and adjacent noncancerous tissues were obtained from patients who undertook surgical resection in Shenzhen Longhua District Central Hospital from 2016 to 2018. These 30 patients did not receive any treatment before surgery and they agreed to sign the informed consent before the operation. These collected tissues were dipped in RNAlater RNA Stabilization Reagent (Qiagen, Hilden, Germany) according to the manufacture’s protocol and then stored in liquid nitrogen for follow-up studies. This study was authorized by the clinical research ethics committee at Shenzhen Longhua District Central Hospital.

### Cell culture

The BEAS-2B immortalized human bronchial epithelial cell line and NSCLC cell lines (A549, NCI-H23, NCI-H358, NCI-H1975 and PC-9) were obtained from American Type Culture Collection (Manassas, VA, USA). BEAS-2B was maintained in BEBM medium (Lonza/Clonetics Corporation, Basel, Switzerland) containing 10% fetal bovine serum (FBS) (HyClone, Logan, UT). NSCLC cell lines were cultured in RPMI-1640 medium (Thermo Fisher Scientific, MA, USA) supplemented with 10% FBS. All cell lines were cultured in a humidified incubator maintained at 37°C in 5% CO_2_.

### mRNA extraction and qPCR assay

Total RNA was extracted from tissues and cells using TRIzol reagent (Invitrogen, CA, USA) according to the manufacturer’s protocol. The synthesis of cDNA was performed with EasyScript One-Step gDNA Removal and cDNA Synthesis SuperMix (Transgen Biotech, Beijing, China) while the reverse transcription of miRNA was conducted using miScript II RT kit (Qiagen). To analyze the relative expression of NOTCH1 mRNA and miR-582-5p, qRT-PCR assay was carried out using 2x SYBR Green qPCR Master Mix (Bimake, Shanghai, China) on a CFX96 real-time thermocycler (BioRad, CA, USA). β-actin and U6 were considered as internal reference for normalizing NOTCH1 and miR-582-5p respectively. The relative expression of NOTCH1 mRNA and miR-582-5p was calculated with the 2^-ΔΔCT^ method. Primers used in this assay were listed as follows: miR-582-5p, forward 5′-GCACACATTGAAGAGGACAGAC-3′ and reverse 5′-TATTGAAGGGGGTTCTGGTG-3′; NOTCH1, forward 5′-CCTAGTTTGGGAGGAGCAGATT-3′ and reverse 5′-GGCATGACACACAACAGACT-3′; β-actin, forward 5′-GACTCATGACCACAGTCCATGC-3′ and reverse 3′-AGAGGCAGGGATGATGTTCTG-5′; U6, forward 5′-GCTTCGGCAGCACATATACTAAAAT-3′ and reverse 5′-CGCTTCACGAATTTGCGTGTCAT-3′.

### Transfection assay

MiR-582-5p mimics, inhibitors and the scramble sequences were purchased from Beijing Ruibo Xingke Biotechnology Co., Ltd. (Beijing, China). pCMV6-NOTCH1 and vector pCMV6-Entry were obtained from Origene Technologies Inc. (Rockville, MD, USA). NSCLC cell lines were plated in 6-well plates, grown to 80% confluence and then transfected with indicated plasmids by using Lipofectamine 2000 (Thermo Fisher Scientific, MA, USA) according to the manufacturer's instruction.

### CCK-8 assay

The transfected cells were seeded into 96-well plates at a density of 800 cells/100 μL per well and cultured overnight. Cell proliferation was measured at different times (0 h, 24 h, 48 h and 72 h) using CCK-8 (Beyotime Biotechnology, Shanghai, China).

### Western blot assay

After transfection, cells were lysed using RIPA lysis buffer (Thermo Fisher Scientific), and then total protein was harvested. The concentration of total protein from different groups was detected using BCA Protein Assay Kit (Thermo Fisher Scientific). After denaturation, 50 μg of protein was loaded into 6% or 8% SDS gel and then blotted on the PVDF membrane (Millipore Sigma, USA). Next, the PVDF membrane was blocked with 5% non-fat milk for 1 h at room temperature, followed by incubate with primary antibodies overnight at 4°C. In the second day, the membrane was exposed to secondary antibodies for 1 h at room temperature. The specific protein bands were visualized by using enhanced chemiluminescence system (Bio-Rad Clarity Western ECL).

### Transwell assay

The invasive potential of NSCLC cells was evaluated by Transwell assay. After transfection, 1.5×10^4^ cells/100 μL were seeded into 24-transwell upper chambers (Corning, NY, USA) of which membranes covered with Matrigel (BD Biosciences) and cultured with no-serum RPMI-1640 medium. RPMI-1640 medium containing 10% FBS was added to lower chambers. After 48 h, upper chambers were dipped in 4% polyformaldehyde for 30 min, followed by 0.1% crystal violet for 30 min at room temperature. Cells on the upper surface of membranes were removed by cotton swabs and invasive cells which moved to the lower surface of membranes were counted under an invert microscope (Olympus). The number of invasive cells in five random fields were counted and then averaged.

### Dual luciferase reporter assay

The wild-type (WT) or mutant (Mut) 3′-UTR sequences of NOTCH1 were synthesized and subcloned into a PsiCheck2 vector respectively (Promega Corporation, WI, USA). Cells were then co-transfected with miR-582-5p mimics and aforesaid plasmids for 48 h using Lipofectamine 2000 (Thermo Fisher Scientific) according to the manufacture’s protocol. Next, firefly luciferase activity and renilla luciferase activity were detected using Dual-Luciferase Reporter Assay System (Promega). Results were shown as the ratio of firefly to renilla luciferase activity.

### Statistical analysis

GraphPad Prism 5.0 software was used to analyze data, which were shown as mean ± SD. The significant difference between two groups was estimated by using Student’s t test while one-way ANOVA was used to evaluate the difference among multiple groups. *P* < 0.05 was regarded as statistically significant.

## Results

### The expression level of miR-582-5p is lower in NSCLC tissues than that in para-carcinoma tissues

**Fig 1:**
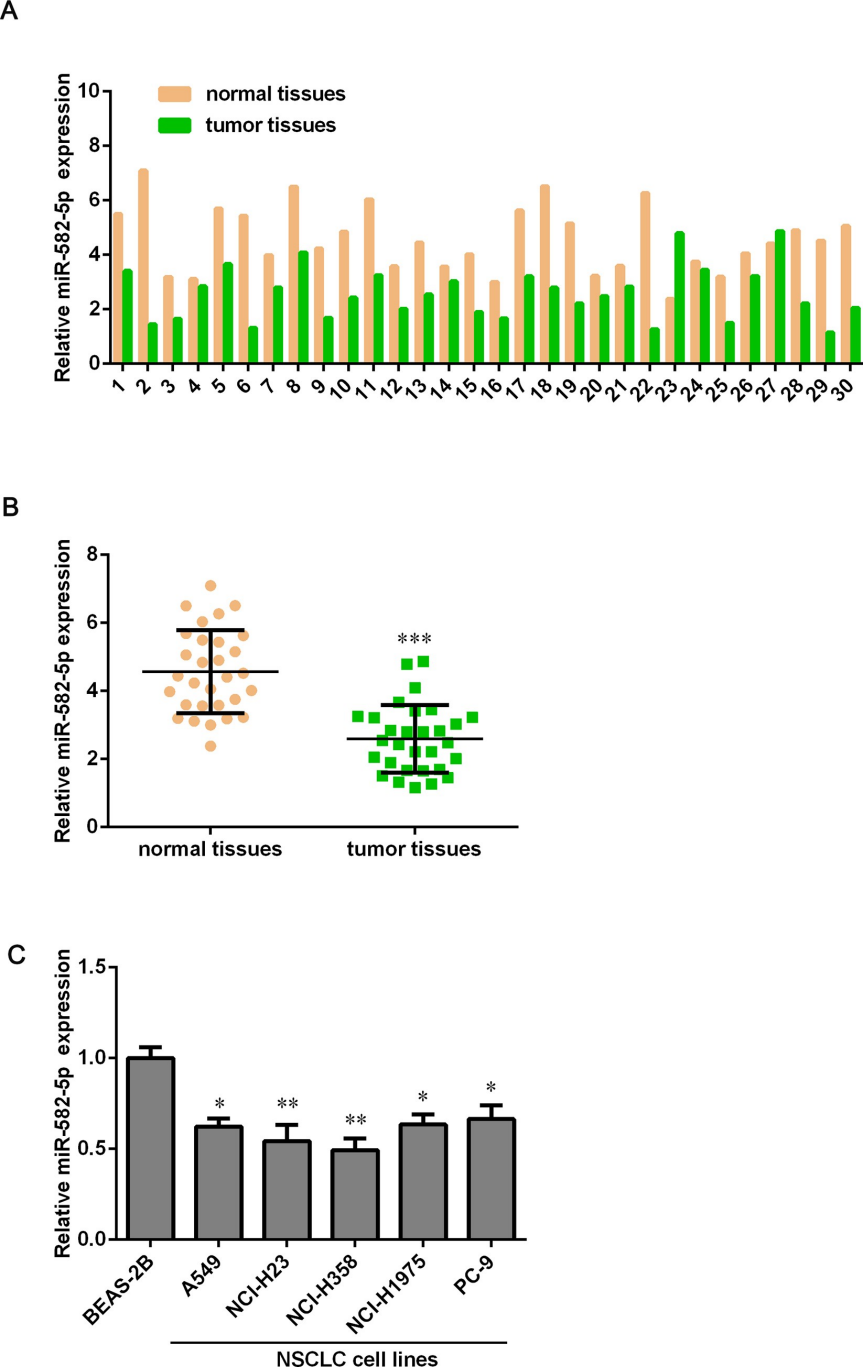
MiR-582-5p is down-regulated in NSCLC tissues compared with para-carcinoma tissue. (A) 30 paired NSCLC tissues and para-carcinoma tissues were collected and the expression level of miR-582-5p in both kinds of tissues was detected by using qPCR assay. (B) The expression of miR-582-5p is markedly lower in NSCLC tissues than that in para-carcinoma tissues. (C) qPCR assay was used to determine miR-582-5p expression in the BEAS-2B immortalized human bronchial epithelial cell line and NSCLC cell lines, including A549, NCI-H23, NCI-H358, NCI-H1975 and PC-9. Data are expressed as mean ± SD. *P<0.05, **P<0.01 and ***P < 0.001 versus the normal tissues’ group or BEAS-2B group.

To understand the potential roles of miR-582-5p in NSCLC, we collected 30 paired NSCLC tissues and para-carcinoma tissues to detect the expression level of miR-582-5p by using qPCR assay. As results shown, there was a significant down-regulation of miR-582-5p expression in NSCLC tissues compared with corresponding adjacent noncancerous tissues, around 1.5-fold decrease (Fig 1A & 1B). To confirm whether miR-582-5p expression is down-regulated both in vitro and vivo, we detected its expression level in NSCLC cell lines using qPCR assay. Surprisingly, we found that miR-582-5p was strongly downregulated in NSCLC cell lines (A549, NCI-H23, NCI-H358, NCI-H1975 and PC-9) compared with the BEAS-2B immortalized human bronchial epithelial cell line (Fig 1C). These results indicated that miR-582-5p might be detrimental to the development and progression of NSCLC.

### Overexpression of miR-582-5p inhibits cell proliferation and invasion

**Fig 2:**
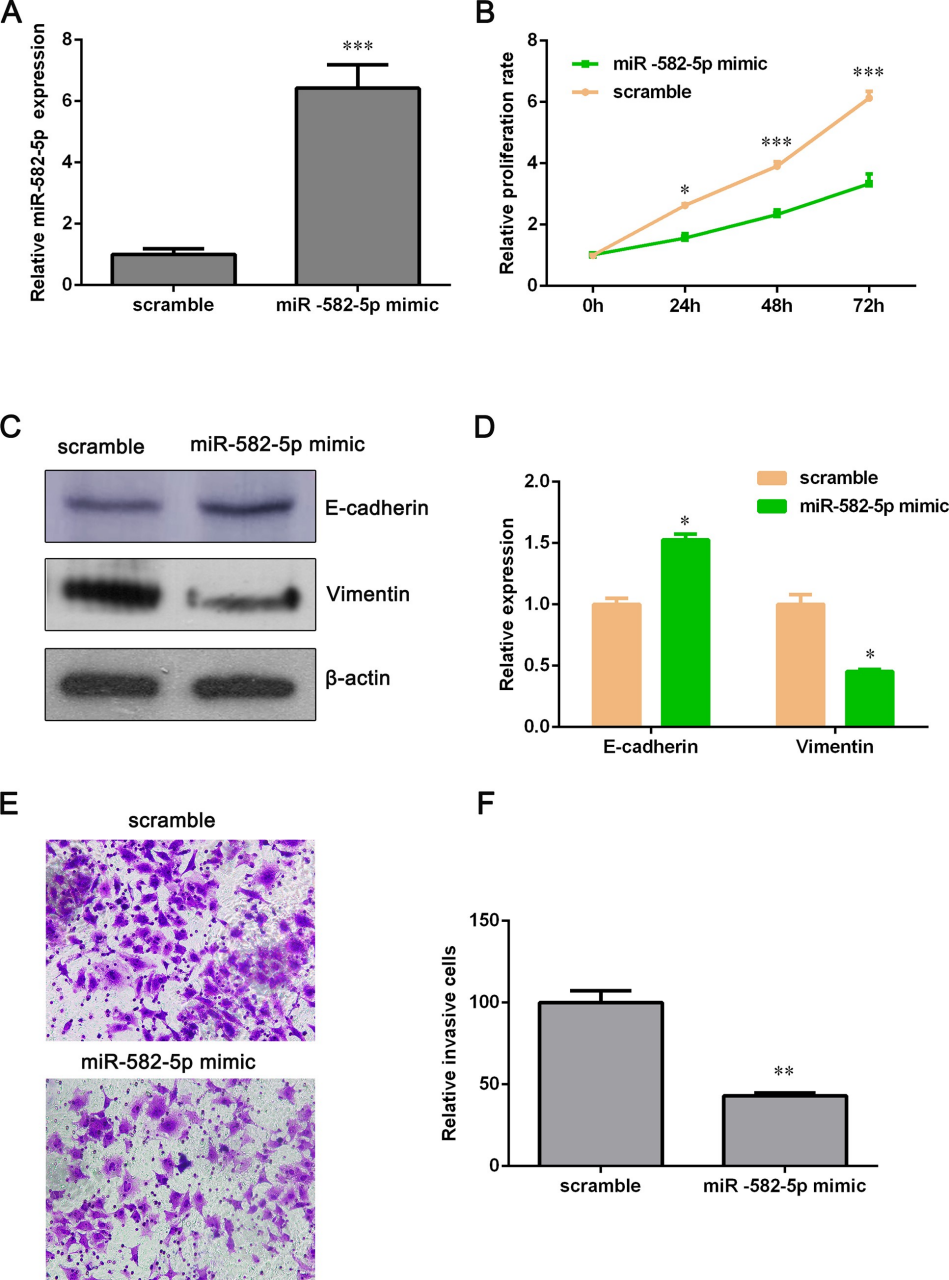
Overexpression of miR-582-5p inhibits proliferation and invasion of NCI-H358 cell. (A) NCI-H358 cells were transfected with miR-582-5p mimics and then miR-582-5p expression was examined with qPCR assay. (B) After transfection with miR-582-5p mimics, NCI-H358 cell proliferation was measured by using CCK-8 assay at different times (0 h, 24 h, 48 h and 72 h). (C) Western blot assay was performed to determine the expression of E-cadherin and Vimentin. (D) Relative expression of E-cadherin and Vimentin was quantified by using Image J software and GraphPad Prism 5.0. (E) Transwell assay was conducted to evaluate the invasive potential of NCI-H358 cells. (F) Relative invasive cells were estimated with Image-Pro Plus tool and GraphPad Prism 5.0. Data are showed as the mean ± SD (n = 3). *P<0.05, **P<0.01 and **P<0.001 versus the scramble group.

From Fig 1C, we can see that the expression of miR-582-5p in NCI-H358 was the lowest among these five NSCLC cell lines. Thus, NCI-H358 cell line was chosen to transfect with miR-582-5p mimics for further investigating the role of miR-582-5p in NSCLC. As shown in Fig 2A, miR-582-5p expression of miR-582-5p mimic group was significantly higher than that of the scramble group after miR-582-5p mimics’ transfection. Next, we examined whether overexpression of miR-582-5p makes an impact on NCI-H358 cell proliferation. As shown in Fig 2B, cell growth was markedly inhibited by overexpression of miR-582-5p. And we observed that ectopic expression of miR-582-5p down-regulated the protein level of Vimentin and up-regulated E-cadherin (Fig 2C and 2D). These two genes are biomarkers of epithelial-mesenchymal transition (EMT), which is associated with cell mobility. Thus, Transwell assay was used to detect the effect of miR-582-5p overexpression on NCI-H358 cell invasion. And the result demonstrated that ectopic expression of miR-582-5p attenuated the invasive capacity of NCI-H358 cell (Fig 2E and 2F). Taken together, these data suggested that overexpression of miR-582-5p suppresses NCI-H358 cell proliferation and invasion.

### Silencing miR-582-5p promotes cell proliferation and invasion

**Fig 3:**
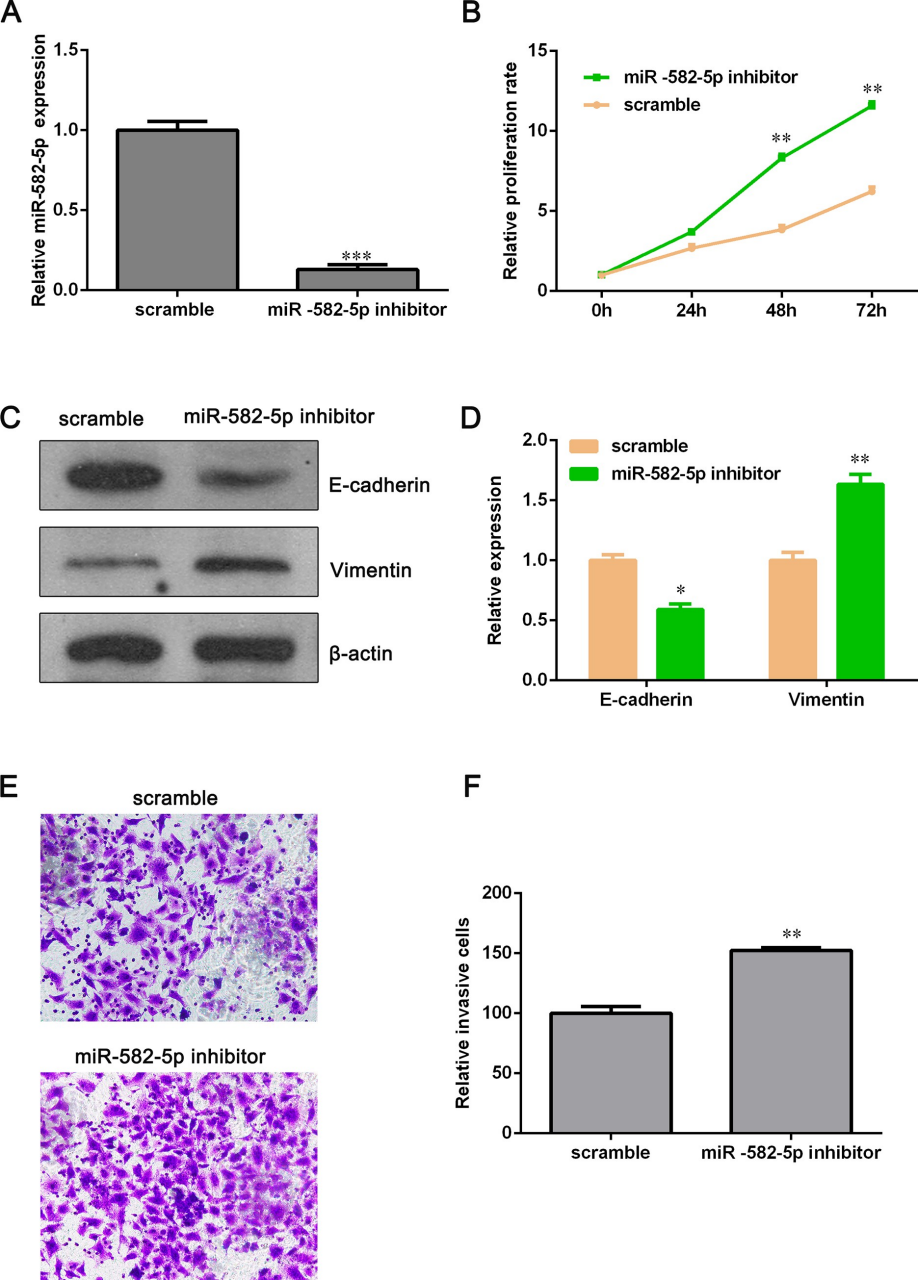
Knockdown of miR-582-5p promotes proliferation and invasion of PC-9 cell. (A) MiR-582-5p inhibitors were transfected into PC-9 cells and the silencing effect was assessed by using qPCR assay. (B) Cell growth rate was measured with CCK-8 assay. (C) The protein level of E-cadherin and Vimentin was detected by Western blot assay after PC-9 cells were transfected with miR-582-5p inhibitors. (D) Relative expression of E-cadherin and Vimentin was analyzed by using Image J software and GraphPad Prism 5.0. (E) The effect on PC-9 cell invasion mediated by miR-582-5p inhibitors was evaluated by Transwell assay. (F) The number of invasive cells was quantified with Image-Pro Plus and GraphPad Prism 5.0. Data are presented as the mean ± SD (n = 3). *P<0.05, **P<0.01 and **P<0.001 versus the scramble group.

To further affirm that miR-582-5p does inhibit NSCLC cell lines’ proliferation and invasion, we silenced miR-582-5p by using miR-582-5p inhibitors. From Fig 1C, miR-582-5p expression in PC-9 cell was higher than that in other four NSCLC cell lines and, hence, PC-9 cell was selected to transfect with miR-582-5p inhibitors. The expression level of miR-582-5p was dramatically decreased by miR-582-5p inhibitors (Fig 3A). But PC-9 cell proliferation was elevated miR-582-5p inhibitors (Fig 3B). Furthermore, we found that epithelial marker, E-cadherin, was decreased and mesenchymal marker, Vimentin, was increased after miR-582-5p inhibitors’ transfection (Fig 3C and 3D). Transwell assay showed that the invasive potential of PC-9 cell was also enhanced by miR-582-5p inhibitors (Fig 3E and 3F). These results indicated that miR-582-5p may serve as a significant suppressor against NSCLC cell lines’ proliferation and invasion.

### MiR-582-5p targets NOTCH1 directly

**Fig 4:**
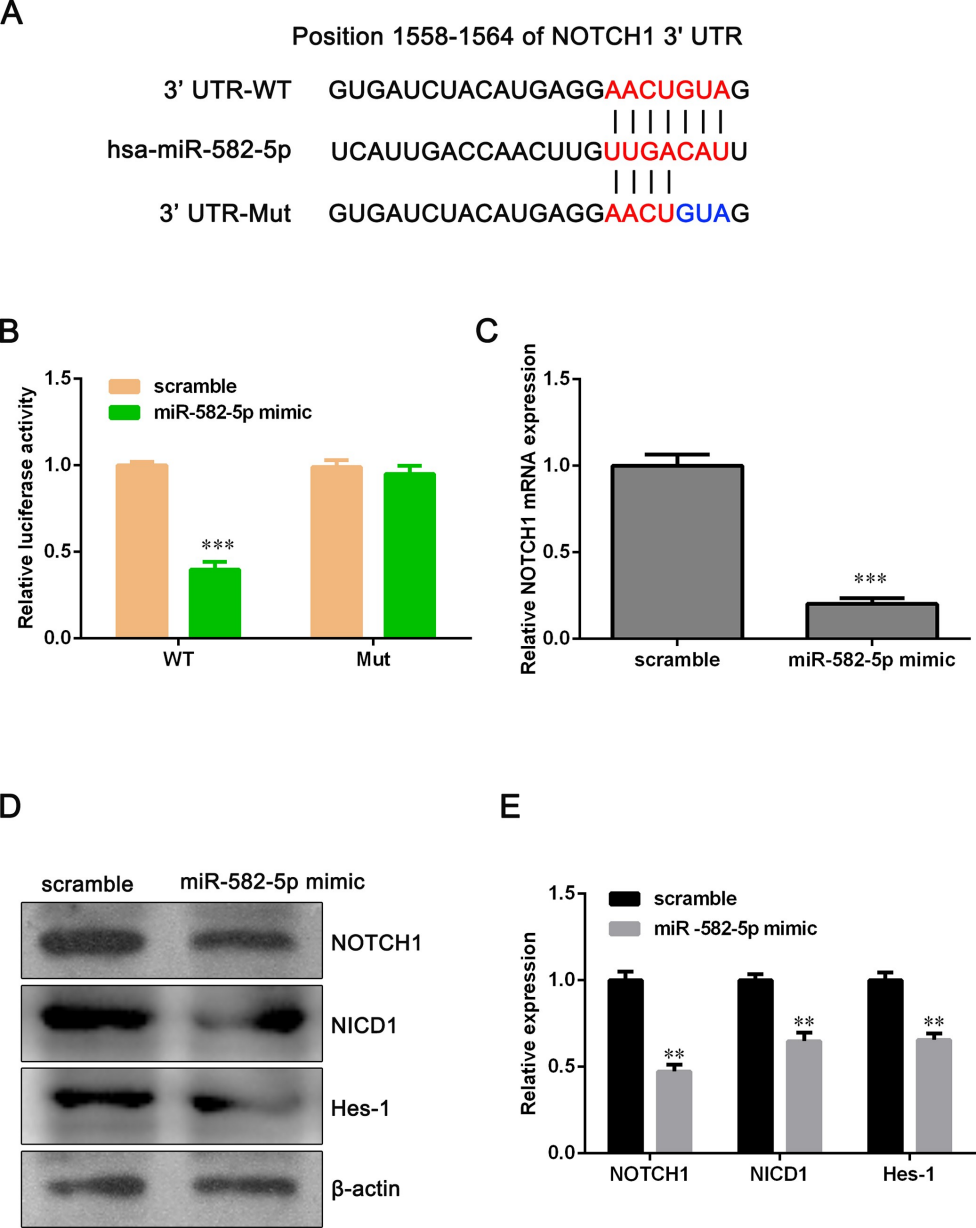
MiR-582-5p directly binds to NOTH1 3’UTR in NCI-H358 cell. (A) Illustration showed the predicted binding site between miR-582-5p and WT-3'UTR-NOTCH1 or Mut-3'UTR- NOTCH1. (B) Dual luciferase reporter assay was conducted to verify the presumptive binding sites. (C) The mRNA level of NOTCH1 was determined with qPCR assay after NCI-H358 cells were transfected with miR-582-5p mimics. (D) The protein level of NOTCH1, NICD1 and Hes-1 was detected by Western blot assay after NCI-H358 cells were transfected with miR-582-5p mimics. (E) Relative expression of NOTCH1 was quantified with Image J and GraphPad Prism 5.0. Data are expressed as the mean ± SD (n = 3). **P<0.01 and ***P<0.001 versus the scramble group.

To explore the molecular mechanism of miR-582-5p function in NSCLC cells, we used TargetScan, PicTar, and miRBase tools to predict functional targets of miR-582-5p and found that NOTCH1 might be a candidate target gene of miR-582-5p. As shown in Fig 4A, miR-582-5p binds to the position 1558–1564 of NOTCH1 3'UTR. To verify this potential direct interaction, dual-luciferase reporter assay was conducted. We firstly constructed luciferase reporter vectors containing the WT or Mut 3'UTR of NOTCH1 and then the WT or Mut luciferase reporter vector was co-transfected with miR-582-5p mimics into NCI-H358 cells. Luciferase activity detection showed that ectopic expression of miR-582-5p reduced the luciferase activity of WT-3'UTR-NOTCH1 significantly but had not inhibitory effect on the luciferase activity of Mut-3'UTR-NOTCH1 (Fig 4B). qPCR and Western blot assay demonstrated that overexpression of miR-582-5p clearly decreased both mRNA and protein level of NOTCH1 (Fig 4C–4E). In addition, we have detected the expression of Notch intracellular domain (NICD1) and Notch target gene, Hes-1. NICD1 and Hes-1 were down-regulated after miR-582-5p mimic transfection, suggesting that miR-582-5p inhibits NOTCH1 pathway (Fig 4D and 4E). These data indicated that NOTCH1 is a target of miR-582-5p.

### The expression level of NOTCH1 is higher in NSCLC tissues than that in para-carcinoma tissues

**Fig 5:**
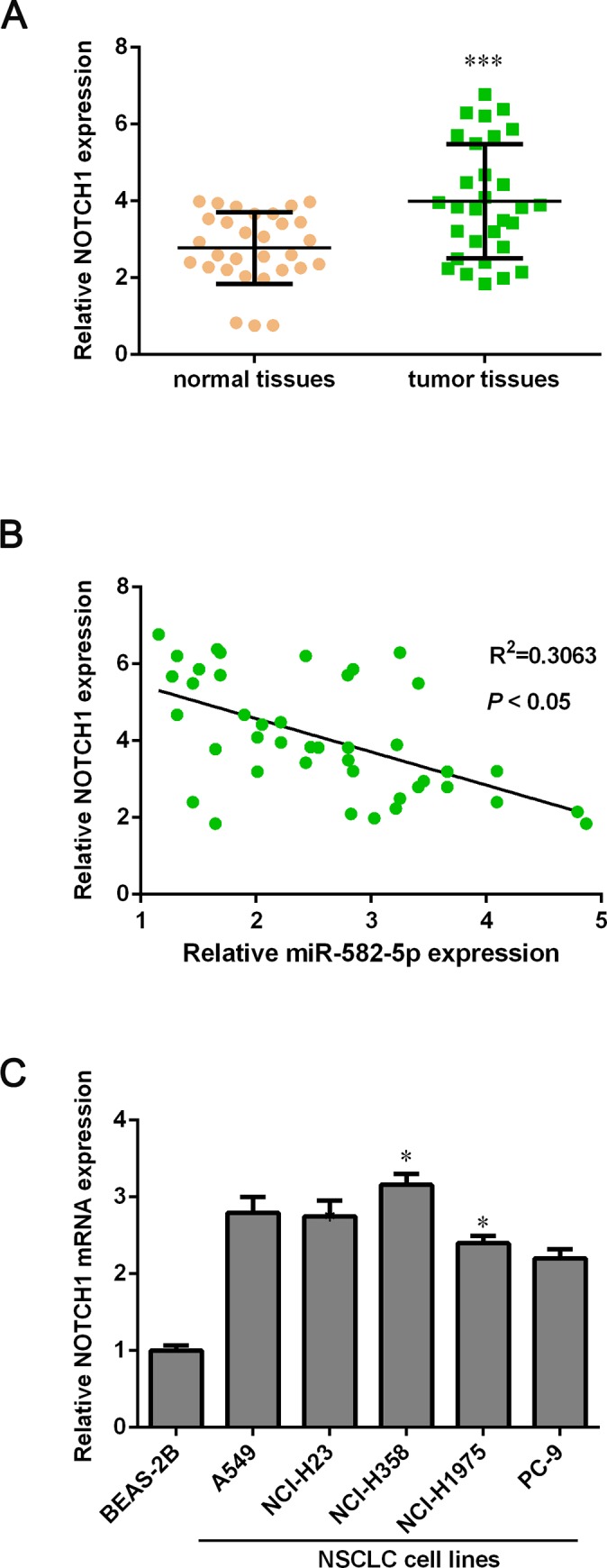
NOTCH1 is up-regulated in NSCLC tissues compared with para-carcinoma tissues. (A) The mRNA level of NOTCH1 in 30 paired NSCLC tissues and para-carcinoma tissues was detected with qPCR assay. The mRNA level of NOTCH1 is significantly higher in NSCLC tissues than that in para-carcinoma tissues. (B) The correlation between miR-582-5p and NOTCH1 is shown. (C) The mRNA expression of NOTCH1 in in the BEAS-2B immortalized human bronchial epithelial cell line and NSCLC cell lines (A549, NCI-H23, NCI-H358, NCI-H1975 and PC-9) was measured by using qPCR assay. Data are expressed as mean ± SD. *P<0.05 and ***P < 0.001 versus the normal tissues’ group or the BEAS-2B cell.

From above results, we reasonably speculated that there might be an inverse correlation between miR-582-5p and NOTCH1. Thus, we detected the mRNA level of NOTCH1 in NSCLC tissues and para-carcinoma tissues by using qPCR assay. As expected, the expression of NOTCH1 was significantly up-regulated in NSCLC tissues compared with para-carcinoma tissues (Fig 5A and 5B). Moreover, we analyzed the relevance between miR-582-5p and NOTCH1 and found that miR-582-5p expression was negatively correlated with the expression of NOTCH1 (*P* < 0.05) (Fig 5C), suggesting that NOTCH1 may play a crucial role in cell growth and invasion inhibition by miR-582-5p.

### Overexpression of NOTCH1 promotes cell proliferation and invasion

**Fig 6:**
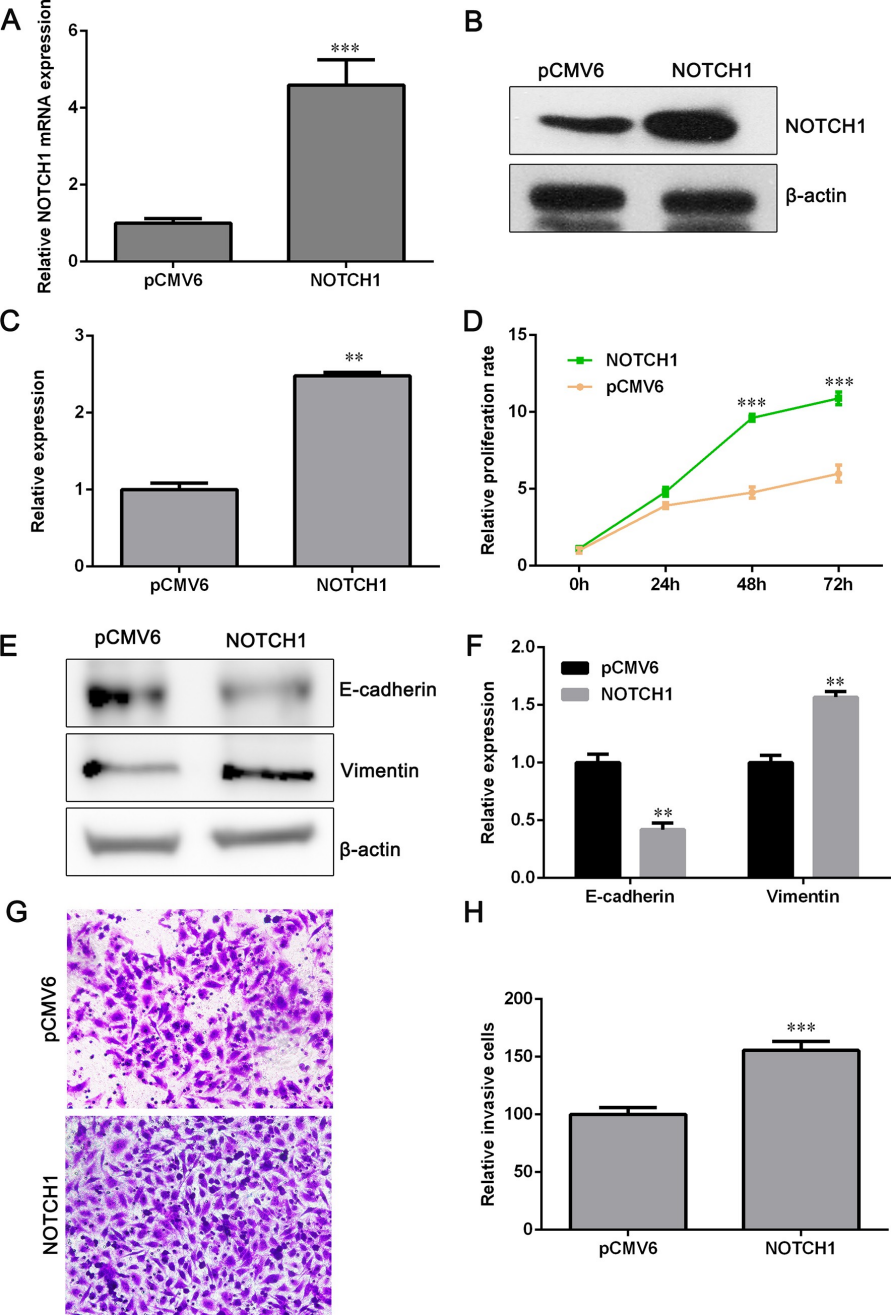
Overexpression of NOTCH1 promotes cell proliferation and invasion. (A) NCI-H358 cells were transfected with NOTCH1 plasmids and the mRNA expression of NOTCH1 was determined with qPCR assay. (B) Western blot assay was performed to detect the expression of NOTCH1 after NCI-H358 cells were transfected with NOTCH1 plasmids. (C) Relative protein expression of NOTCH1 is shown. (D) Cell proliferation was detected by CCK-8 assay after NCI-H358 cells were transfected with NOTCH1 plasmids or pCMV6 vectors. (E) The protein level of E-cadherin and Vimentin was assessed by Western blot assay. (F) Relative protein expression of E-cadherin and Vimentin is shown. (G) Transwell assay was used to determine cell invasive ability. (H) Relative number of invaded cells is shown. Data are shown as mean ± SD (n = 3). **P<0.01 and ***P<0.001 versus the pCMV6 group.

Since higher expression of NOTCH1 was observed in NSCLC tissues and cell lines than that in para-carcinoma tissues and human bronchial epithelial cell line BEAS-2B, we next examined the impact of NOTCH1 overexpression on NSCLC cell growth and invasion. qPCR and Western blot assays showed that the mRNA and protein level of NOTCH1 were up-regulated dramatically after transfection of NOTCH1 overexpression plasmids (Fig 6A–6C). Ectopic expression of NOTCH1 significantly increased NCI-H358 cell proliferation rate (Fig 6D). The expression of E-cadherin was reduced and Vimentin was elevated after cells were transfected with NOTCH1 overexpression plasmids (Fig 6E and 6F). Furthermore, cell invasive capacity was enhanced by NOTCH1 overexpression (Fig 6G and 6H), indicating that NOTCH1 has a positive effect on NSCLC cell EMT.

### Overexpression of NOTCH1 antagonizes miR-582-5p inhibitory effect on NSCLC

**Fig 7:**
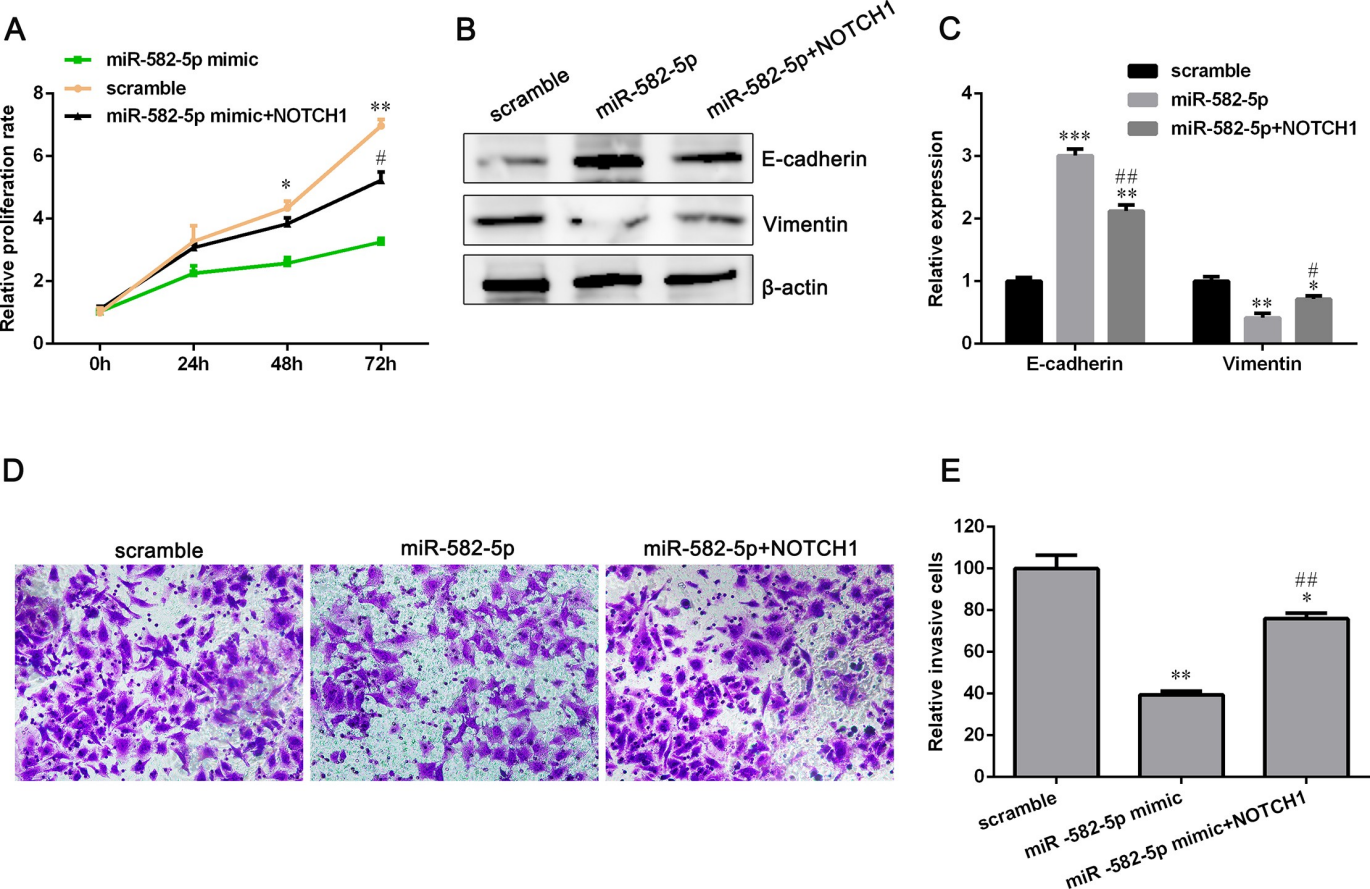
Overexpression of NOTCH1 rescues the miR-582-5p-mediated inhibition in NCI-H358 cell proliferation and invasion. (A) NCI-H358 cells were co-transfected with miR-582-5p mimics and NOTCH1 plasmids or vectors. And cell proliferation was assessed by CCK-8 assay. (B) Western blot assay was applied to examine EMT markers’ expression. (C) Relative protein expression of E-cadherin and Vimentin is shown. (D) Cell invasion was evaluated with Transwell assay. (E) Relative number of invaded cells is shown. Data are presented as mean ± SD (n = 3). *P<0.05, **P<0.01 and ***P<0.001 versus the scramble group. ^#^*P*<0.05, ^##^*P*<0.01 versus the miR-582-5p mimic group.

The mRNA level of NOTCH1 was the highest in NCI-H358 cell among the five NSCLC cell lines (Fig 5C). However, miR-582-5p expression was the lowest in NCI-H358 cell compared with other four NSCLC cell lines (Fig 1C). Therefore, NCI-H358 cell was chosen to perform a rescue experiment to determine whether miR-582-5p inhibited NSCLC cell lines proliferation and invasion via targeting NOTCH1. NCI-H358 cells were co-transfected with miR-582-5p mimics and NOTCH1 overexpressing plasmids. The proliferation of NCI-H358 cells with miR-582-5p overexpressing was partially increased after transfected with NOTCH1 plasmids (Fig 7A). Compared with miR-582-5p mimics group, the expression of E-cadherin was down-regulated and Vimentin was up-regulated in co-transfected group, indicating that NOTCH1 can reverse EMT inhibition caused by miR-582-5p overexpression (Fig 7B and 7C). Transwell assay showed that the invasive ability of NCI-H358 cells co-transfected with NOTCH1 plasmids and miR-582-5p mimics was enhanced compared with those cells co-transfected with vectors and miR-582-5p mimics (Fig 7D and 7E). To sum up, these results implied that miR-582-5p suppresses NSCLC cell proliferation and invasion through down-regulating NOTCH1.

## Discussion

Increasing evidence demonstrate that miRNAs take part in tumorigenesis and progression via targeting tumor suppressive genes or oncogenes, which are associated with cancer cell growth, migration, invasion, angiogenesis and apoptosis[21]. Previous studies demonstrated that miR-582-5p exhibits different effects against different kinds of cancers. In human endometrial carcinoma, up-regulating the expression of miR-582-5p could inhibit cell proliferation and induce apoptosis. Further mechanism study showed that miR-582-5p exerted the inhibitory effect through targeting AKT3[22]. Study by Wang et al described that miR-582-5p suppressed metastasis of salivary adenoid cystic carcinoma cells via targeting FOXC1[9]. In gastric cancer, miR-582-5p has been verified to restrain cell growth by down-regulating the expression of AKT3[23]. Uchino et al clarified that miR-582-5p and miR-582-3p functioned effectively in the inhibition of bladder cancer progression[24]. However, D.H. et al proved that miR-582-5p characterized anti-apoptotic function, promoting human glioblastoma stem cell survival via directly inhibiting caspase 3, caspase 9, and Bim[25]. Study by Maeno et al demonstrated that up-regulation of miR-582-5p facilitated cellular proliferation of prostate cancer cells under androgen-deprived conditions[11].In colorectal cancer, miR-582-5p has showed to elevate cell growth rate by targeting adenomatous polyposis coli (APC)[26]. The exact function of miR-582-5p in NSCLC remains unknown.

In the present study, we firstly investigated the expression level of miR-582-5p in NSCLC tissues and pericarcinomatous tissues. Surprisingly, miR-582-5p was down-regulated in NSCLC tissues compared with para-carcinoma tissues. We have analyzed the correlation between miR-582-5p expression level and clinical features in these 30 NSCLC patients. As shown in S1 Table, high miR-582-5p expression was negatively correlated with tumor size (> 3 cm), advanced tumor stage (Ⅲ, Ⅳ) and tumor metastases, implying that miR-582-5p might be a tumor suppressor. The expression level of miR-582-5p was also lower in NSCLC cell lines than that in the BEAS-2B immortalized human bronchial epithelial cell line, indicating that miR-582-5p might be a tumor suppressor for NSCLC. To explore the biological function of miR-582-5p in NSCLC, NSCLC cell lines were transfected with miR-582-5p mimics and inhibitors respectively. Overexpression of miR-582-5p suppressed cell proliferation and invasion while knockdown of miR-582-5p promoted cell growth and metastasis, suggesting miR-582-5p serves as a suppressor in NSCLC.

Deregulated NOTCH signaling with gene mutations or aberrant expression of NOTCH receptors has been reported in solid tumors, including NSCLC[27, 28]. Accumulating evidences showed that NOTCH1 is implicated with the proliferation and invasion of NSCLC. Study by Wael et al demonstrated that NOTCH1 signaling regulated cell growth, apoptotic cell death and differentiation of lung cancer. Huang et al[29] found that NOTCH1 activation helped an oncogenic protein, Thymosin beta 4, contribute cell proliferation and invasion in NSCLC. Licciulli et al[30] found that NOTCH1 function was required for the initiation of lung adenocarcinoma, belonging to the histopathologic class of NSCLC, and controlled tumor cell survival via suppression of p53-mediated apoptosis through the regulation of p53 stability. Recent study by Xu et al showed that KIAA0247 restrained NSCLC progression through attenuating proliferation, migration and invasion of cancer cells through inhibition of the NOTCH pathway[31]. Clinically oriented studies have highlighted that NOTCH signaling impacts survival in lung cancer patients. Through evaluating the relationship between prognosis and the expression level of NOTCH ligands and receptors in NSCLC, Donnem et al found that higher NOTCH1 expression indicated poorer outcomes in lung adenocarcinoma statistically significantly[32]. These studies supported a strong and direct role of NOCTH signaling in NSCLC.

In our study, we assessed the expression of NOTCH1 in NSCLC tissues and para-carcinoma tissues respectively and found that NOTCH1 was down-regulated in NSCLC tissues, which was accordant with previous studies. Furthermore, there was a negative relationship between NOTCH1 and miR-582-5p. We also found that the mRNA and protein level of NOTCH1 was decreased by overexpression of miR-582-5p. In our research, miR-582-5p showed inhibitory effect on NSCLC cell lines’ proliferation and invasion. Since NOTCH1 has been proven to play an important role in NSCLC cell survival and metastasis, we reasonably speculated that NOTCH1 may be implicated with the cell proliferation and invasion inhibition by miR-582-4p.

NOTCH signaling pathway modulates the expression of those proteins which are important for the establishment of cell-cell connections and cell polarity[33]. Using a γ-secretase inhibitor to inactivate NOTCH signaling can reverse the EMT process, from motile and invasive mesenchymal phenotypes to polarized epithelial properties[34]. Loss of E-cadherin is not conducive to cell adhesion, causing destabilization of the epithelial structure. It is a primal process in EMT. The expression level of Notch-ICD has been proven to be associated with E-cadherin. Ectopic expression of NOTCH-ICD elevated snail expression and thus decreased the expression of E-cadherin. By contrast, inactivation of NOTCH reduced snail expression, leading to increasing E-cadherin expression, indicating that a direct role for NOTCH signaling in the induction of EMT[35]. In addition, inactivation of NOTCH signaling attenuated the expression of mesenchymal markers, including snail, slug, ZEB1 and vimentin, resulting in partial reversal of EMT[36]. In this study, overexpression of NOTCH1 partially weakened the inhibition of cell proliferation and invasion by miR-582-5p in NSCLC cell lines, suggesting that miR-582-5p restrains cell growth and the invasive potential of NSCLC by down-regulating NOTCH1. In conclusion, our findings revealed an inhibitory role for miR-582-5p via targeting NOTCH1 in NSCLC.

## Supporting information

S1 Table Supplementary Table 1. (DOCX)
