# Supplementary material for: MicroRNA-582-5p suppresses non-small cell lung cancer cells growth and invasion via downregulating NOTCH1
Source: PLoS One. 2019 Jun 6;14(6):e0217652. doi: 10.1371/journal.pone.0217652 (PMC6553855; doi:10.1371/journal.pone.0217652)
Supplement: S1 Table — (DOCX) [file pone.0217652.s001.docx]

Supplementary Table

**Supplementary Table 1. Correlation between miR-582-5p expression**

**and the** **30 NSCLC patients’ clinicopathological parameters**

| Parameters | MiR-582-5p expression | | P-value |
| --- | --- | --- | --- |
|  | High (n=15) | Low (n=15) |  |
| Age |  |  | 0.7152 |
| ≤ 55 | 7 | 9 |  |
| > 55 | 8 | 6 |  |
| Sex |  |  | 0.7104 |
| Male | 10 | 8 |  |
| Female | 5 | 7 |  |
| Tumor size |  |  | 0.0268 |
| ≤ 3 cm | 11 | 4 |  |
| > 3 cm | 4 | 11 |  |
| TNM stage |  |  | 0.0253 |
| Ⅰ, Ⅱ | 12 | 5 |  |
| Ⅲ, Ⅳ | 3 | 10 |  |
| Metastasis |  |  | 0.0025 |
| Yes | 2 | 11 |  |
| No | 13 | 4 |  |

P-value was analyzed with a two-sided Pearson chi-square test.
